# Participants’ perceptions of interactions with community health workers who promote behavior change: a qualitative characterization from participants with normal, depressive and anxious mood states

**DOI:** 10.1186/s12939-018-0729-9

**Published:** 2018-02-05

**Authors:** Joseph Perales, Belinda M. Reininger, MinJae Lee, Stephen H. Linder

**Affiliations:** 1 La Clínica – Casa del Sol, 1501 Fruitvale Ave, Oakland, California 94601 USA; 2 UT Health School of Public Health in Brownsville, Division of Health Promotion & Behavioral Sciences and Hispanic Health Research Center, One West University Blvd, Brownsville, TX 78520 USA; 3 Michael & Susan Dell Center for Healthy Living, UT School of Public Health, Austin Regional Campus, University of Texas Administration Building (UTA), 1616 Guadalupe Street, Suite 6.300, Austin, Texas 78701 USA; 4 University of Texas Health Science Center at Houston, Center for Clinical and Translational Sciences, 7000 Fannin, Suite 1800, Houston, Texas 77030 USA; 5 University of Texas Health Science Center at Houston, Biostatistics/Epidemiology/Research Design (BERD) Core, Center for Clinical and Translational Sciences, 7000 Fannin, Suite 1800, Houston, Texas 77030 USA; 6 UT Health School of Public Health, Institute for Health Policy, Division of Management, Policy and Community Health, 6410 Fannin, Houston, TX 77030 USA

**Keywords:** Community health worker, Latino, Mental health, Depression, Anxiety, Intervention

## Abstract

**Background:**

Interventions that promote healthier lifestyles among Latinos often involve community health workers (CHWs). CHWs can effectively advocate for healthier lifestyles and may be pivotal in addressing such mental health conditions as depression and anxiety. The goal of this study was to characterize the relationship dynamics between Latino participants and CHWs, from the participant’s perspective. We aimed to determine if CHW-delivered community interventions effected behavior change, especially among participants who reported anxiety and depression.

**Methods:**

Semi-structured interviews were conducted with a purposive sample of 28 Latino participants that was based on a mental health scoring strata. Participants completed a lifestyle intervention that included multiple home visits from CHWs to promote physical activity and healthful food choice. Interviews were conducted in the participant’s preferred language (English or Spanish). Transcribed interviews were analyzed using a grounded theory approach until concept saturation was achieved.

**Results:**

The sample was primarily female (82%), lower socioeconomic status (64%), and mean age of 50 years. Participants discussed the rapport building and professionalism of CHWs as a feature that facilitated strong, positive relationships and lifestyle behavior changes. Participants described how CHWs patterned their change approaches, which were similar to commonly used therapeutic techniques in the treatment of anxiety and depression. While anxiety and depression were described as having an impact on behavior change, most, but not all, participants who reported negative mood states said that the CHW relationship helped in changing that state to some extent.

**Conclusions:**

Participants’ perceptions indicated that positive personal changes were influenced by CHWs. Only participants who reported consistently poor scores for depression, anxiety or both reported negative or neutral experiences with the CHWs. This study lends qualitative support to the use of CHWs as extenders of care, particularly in areas that have a shortage of primary and mental health care providers.

## Background

According to U.S. Census Bureau projections, the Latino population, including foreign-born individuals, will rise to 119 million by 2060 [1]. As Latino population concentrations rise, so does the prevalence of health disparities [2], including disparities in their mental health treatment. Research indicates that compared with their white counterparts, Latinos, especially immigrant populations, are far less likely to receive treatment for mental illness [3, 4]. Exacerbating these disparities is the shortage of mental health care workers nationally [5–7], and it is even more pronounced along the US-Mexico border [8, 9]. With this shortage, Latinos who need services may not be offered them, may have to wait months and years to receive them, or once offered may receive truncated services. In addition, Latinos’ cultural components of identity may contribute to their underutilizing mental health services. We see continued dependence on community, family, and traditional healers as limiting participation in formal systems of care [10]. These practices can inhibit diagnosis, and as a result, depression is often underdiagnosed among Latinos or misattributed to other etiologies, like temporary social strains or stressors [11, 12]. Community health workers (CHWs) may hold some capacity to effectively address specific mental health needs within Latino populations [13].

CHWs have worked effectively with Latinos because they are often members of the communities they serve, and they understand the ethnicity, language, values, socioeconomics, coping, and traditional practices that impact life experiences in their geographic areas [14–17]. Moreover, CHWs have been linked with improved chronic disease health outcomes [17–28], consistently demonstrated in diabetes self-management education and training [17–21], cholesterol screenings [29], tobacco use cessation [30], hypertension and cardiovascular disease management and control [22–24] as well as in other chronic disease interventions [25, 27]. Israel [31] wrote extensively about CHWs providing support within social networks as a framework for improving health outcomes in communities. CHWs have been effective in increasing their priority population’s internal sense of empowerment, perceived control over decision making, competence within the community, problem-solving strategies, and personal reduction of stressors [31].

Alongside the growing body of literature on how CHWs can help improve health outcomes, there is research that supports the role of lay counselors [32–35] and consumers/peers in addressing mental health outcomes [36]. While studies note the growth across continents, they also describe the need to tailor the lay counselors’ approaches to local cultures [37] and to coach and supervise peers [38]. Many researchers and practitioners are calling for greater attention to dissemination and implementation efforts of lay counselors for mental health care in low resource communities and countries [39]. Recent trials have shown that task-shift approaches, wherein lay counselors with short, intense training, have similar positive patient outcomes as do highly trained professional counselors [32].

Despite the positive counseling role that CHWs may play, there has been little research on the CHW role in behavioral change and the therapeutic effects of that change, particularly among individuals with anxiety or depression. Therefore, the purpose of this study was to characterize Latino participant perceptions of their interactions with CHWs who delivered a community intervention in order to understand the dynamics of that relationship and the behavior change, particularly among participants who reported anxiety and depression.

## Methods

### Research design

We conducted individual, in-depth, semi-structured interviews with a sample of Latino participants who previously took part in a CHW-delivered behavioral intervention.

### Parent study

Interview participants were recruited from the intervention arm (*n* = 250) of a behavioral intervention trial testing the *Tu Salud* ¡*Si Cuenta!* (TSSC) *Your Health Matters! at Home* outreach modules delivered by CHWs. Specifically, the intervention tested whether six CHW-delivered educational and motivational outreach modules and social support improved healthy food choices and physical activity outcomes. The modules were developed over a 2-year period with both CHW and community stakeholder input throughout the development, testing, and redevelopment cycle.

Each module promotes discussion about behavior change using motivational interviewing strategies, video-based role-model stories, goal-setting activities and key facts. Additionally, the intervention arm content addressed basic human needs like companionship, intimacy, sense of belonging, and self-esteem, all linked to enhanced well-being and health [40]. The content related to physical activity is rooted in evidence that increased physical activity is protective and can mitigate certain mental health conditions, namely anxiety and depression [41–51].

The intervention was delivered by 8 bilingual (Spanish/English) CHWs working in pairs between baseline and the 6-month follow-up. All CHWs were female; four have worked as CHWs with a University for at least 3 years and the other 4 were new to the University and to the CHW role. Three CHWs had bachelor degrees from their home country, Mexico, four had high school education, and one had less than a high school education. Throughout the intervention the CHWs’ deliveries were monitored by a program manager who supported them with consultations on strategies to support participants (referrals, behavioral change theory discussions and strategies for building on cultural beliefs).

The behavioral intervention trial included assessments at baseline, 6 and 12 months for anxiety and depression assessments using the Center for Epidemiological Studies Depression (CES-D) scale and the Zung Self-Rating Anxiety scale (SAS) [52, 53]. CHWs were unaware of participants’ anxiety and depression scores.

### Setting

This study was conducted in Cameron County, Texas, on the Texas-Mexico border, in an area known as the Rio Grande Valley. The area’s ethnic distribution is 88.1% Latino, of which 80.5% are Mexican-American, and 34.8% of the county’s population, or just over 400,000 individuals, live below the poverty level [54]. Some 65.1% of the county’s residents are without health insurance, and 84.2% are obese (50.9%) or overweight (33.3%) [55]. Approximately 85% did not meet the recommended guidelines for the daily consumption of fruits and vegetables, 66.7% did not meet the recommended levels of physical activity, 16.2% were considered to be smokers and 4.7% were heavy alcohol users [56]. Within this population, the three leading causes of death are heart disease, cancer and stroke [57].

### Qualitative study design

The present sub-study is a qualitative investigation to better understand the dynamics of the CHW-participant relationship and of behavior change and, particularly, the role anxiety and depression may play. This sub-study was conducted after the parent behavioral intervention trial was complete, and to avoid bias, the CHWs who delivered the intervention did not collect data.

### Sampling and sample size

A list of potential participants was developed based on the criteria described below, and recruitment was conducted via phone by the study staff to determine interest. The study staff consisted of four research assistants culturally, linguistically, and ethnically similar to the study participants were recruited. The three females and one male were college educated and held full- or part-time professional positions at the University in which their main roles involved community interaction. The research assistants all received extensive training on the instrument, techniques for probing, and use of recording technology. Additionally, protocols for naming systems, privacy when transporting participant information, and reaffirming informed consent were part of their training.

**Fig. 1:**
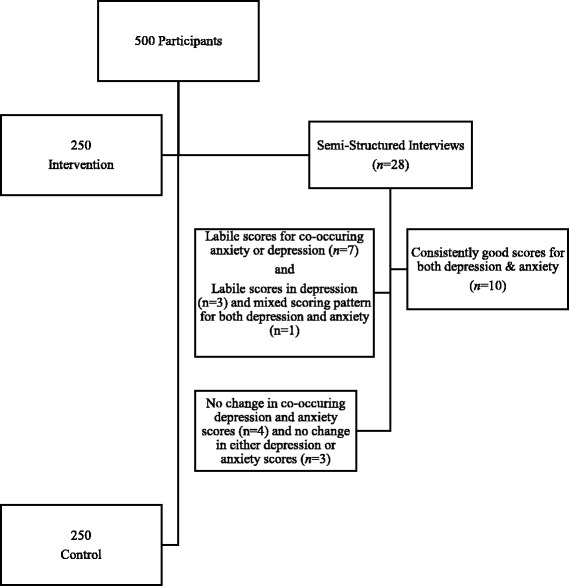
Description of sample mental health scoring strata

The purposive sample of 28 Latino participants was drawn from the intervention arm of the TSSC behavioral intervention trial. To avoid time and recall bias, participants contacted about the study were prioritized based on their having seen a CHW within the previous 9 months. Additionally, three mental health scoring strata were used to recruit individuals who represented those strata, with the goal of having at least half the sample reporting anxiety and/or depression scores above normal levels (Fig. 1).

A more balanced sample was planned by gender; however, the majority were female because women were more likely to agree to participate. If a participant was interested and met the criteria, a meeting was scheduled during which the study staff confirmed eligibility and reviewed the consent form in the language of choice and, if the participant gave consent, proceeded with the interview.

### Sociodemographic variables

The following characteristics were collected at baseline: sex, age, SES quartile (first quartile income ≤$17,830; second quartile > $17,830 and ≤ $24,067; third quartile > $24,067 and ≤ $31,747; fourth quartile >$31,747), educational attainment (< 8 years, did not complete middle school, 8 years, completed middle school or more), height, weight, level of physical activity, fruit and vegetable consumption, health insurance (insured or not insured), diabetes status (meets American Diabetes Association [58] definition or not).

### Instrument and data collection

The interviews followed a 12 question guide of open- and closed-ended questions that allowed for free flowing conversation and participant responses. The closed-ended questions allowed the participant to classify his/her initial response and then to elaborate on that response through the open-ended probes. The guide was pre-tested by conducting interviews with participants demographically similar to those in the parent study (from which the qualitative study sample was drawn) but who were recruited from community-based settings where CHWs held classes. Based on the pre-test interviews with 17 participants and a series of follow-up questions to assess understanding, minor changes to the questions were made in language and wording. The pre-testing process also resulted in the guide’s final structure.

### Semi-structured interview process

The interviews were conducted in participants’ homes, one-on-one in the participant’s language of choice, 22 in Spanish and 6 in English. Each interview took no more than 30 min and was recorded for accuracy. Interviewers took notes on each question. These were later compared to the recording transcripts to ensure fidelity and accuracy of the audio data.

### Data analysis

We followed a grounded theory approach for qualitative data management and analysis. In this way, data analysis began simultaneously with data collection to inform later data collection efforts. We recorded all interviews and used a professional company to immediately translate and transcribe the audio recordings. The lead author (I.J.P.) reviewed and ensured accuracy of transcripts. The final transcripts were uploaded to Atlas.ti software for analyses [59], identification, and memo development [60]. The simultaneous interviewing/analysis process allowed for research assistants to be given feedback about their interview techniques, including slowing down the interview pace, describing the intervention in more detail to help the participant recall the experience with the CHW, and incorporating minor modifications to words used in the questions. This cycle also ensured that interviews were not terminated until construct saturation was achieved.

**Fig. 2:**
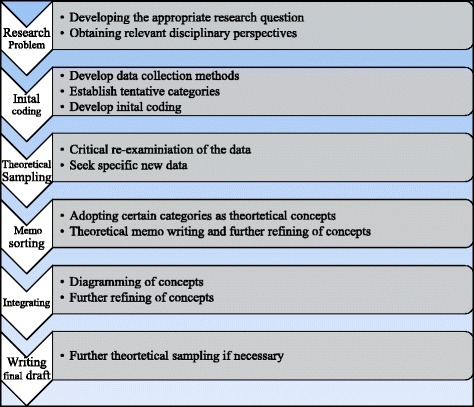
Data analysis procedure

The construction of grounded theory involves identifying codes that anchor the qualitative data and are further developed into concepts and categories that thematically reveal the subject of the research. We applied these methods in the steps detailed in Fig. 2. The transcripts were read for content and coded for themes that were based on the content. These were then assigned to four hierarchical levels of meaning. The data were abstracted multiple times and analyzed using methods recommended for constructing grounded theory [60]. Author I.J.P. conducted the initial coding, and B.M.R. reviewed and validated the codes. Agreement between the two coders existed, but there was discussion about themes and relationships between themes. In these discussions, the two coders reviewed the quotes until consensus was reached.

As part of the qualitative analysis, we further examined the participants with labile scores. The process began by reclassifying the labile scorers according to the distribution of their scoring patterns. These were monotonic increasing, monotonic decreasing, and peaked scorers. Monotonic increasing scorers were those that progressively exhibited overall improvements in mood states. Monotonic decreasing scorers exhibited poorer mood states at the last measurement. Lastly, some labile scorers displayed peaked scoring patterns, that is, they experienced a change at the 6-month time point.

## Results

Most of the 28 participants were middle-age females from the lower socioeconomic strata and had lower education levels. Only 2 (7.1%) individuals met the US Federal daily nutritional requirements for fruits and vegetables consumption [61], and 9 (32.1%) met the US Federal physical activity weekly guidelines of 150 min per week of moderate intensity physical activity [62]. The mean body mass index was 32.43, the mean depression score (CES-D) was 13 and anxiety score (ZUNG SAS) was 35. Of the 28 participants, 18 reported depression or anxiety scores about the normal range (Table 1).

**Table 1:** Description of sample (*n* = 28) based on either 12-month time point measures or change across the 12 months

Male *n* (%)	5 (17.9%)
Female *n* (%)	23 (82.1%)
Age in years *n* (%)	50 (13.421)
< 20	0
20–29	1 (3.6%)
30–39	5 (17.9%)
40–49	8 (28.6%)
50–59	7 (25%)
60–69	4 (14.3%)
≥ 70	3 (10.7)
SES *n* (%)	
First quartile (lowest income)	4 (14.3%)
Second quartile	6 (21.4%)
Third quartile	14 (50%)
Fourth quartile	4 (14.3%)
Education >8th grade, *n* (%)	21 (75%)
Height in cm mean (SD)	158.92 (9.02)
Weight in kg mean (SD)	81.91 (21.77)
Met Physical Activity Weekly Guidelines *n* (%)	9 (32.1%)
Met Daily Fruit/Vegetable consumption Guidelines *n* (%)	2 (7.1%)
Insurance *n* (%)	10 (35.7%)
^a^Diabetes *n* (%)	5 (17.9%)
CES-D Depression Score, median (IQR)	13 (3–21.5)
SAS Anxiety Score, median (IQR)	35 (26–45.75)
BMI mean (SD)	32.43 (7.83)
Underweight *n* (BMI < 18.5)	0
Normal *n* (BMI ≥ 18.5 and < 25)	3 (10.7%)
Overweight *n* (BMI ≥25 and < 30)	11 (39.3%)
Obese *n* (BMI ≥ 30 and < 40)	10 (35.7%)
Morbidly Obese *n* (BMI ≥ 40)	4 (14.3%)

*SD* standard deviation, *SES* socioeconomic status, *CES-D* Center for Epidemiological Studies Depression scale, *SAS* Zung Self-Rating Anxiety scale, *BMI* body mass index, *IQR* interquartile range (25th, 75th)

^a^Diabetes diagnosis, receiving medication to control diabetes or diabetes care

**Fig. 3:**
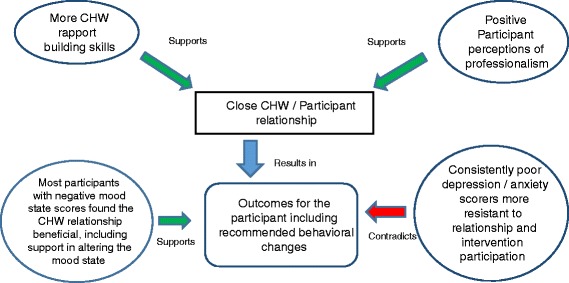
Dynamics of CHW / participant relationship

Based on the grounded theory approach to analysis, themes emerged from the data that elucidated participant perceptions of their interactions with CHWs (Fig. 3), behavior change and any therapeutic effects among individuals with high anxiety and depression scores.

### Participant-CHW relationship

Establishing and building a relationship between the participant and the CHW was influenced by the CHW’s rapport-building skills and the participant’s perceptions of the CHW’s professionalism. According to the participants, these aspects influenced the closeness of the relationship, which, in turn, influenced participants’ willingness to engage with the intervention and to modify their behaviors. Moreover, these aspects were not independent but they did interact, in that they captured the technical knowledge and skills CHWs brought to the relationship.

The participants gave specific examples of effective rapport-building strategies including perceived level of CHW support, if the CHW offered any self-disclosure, the validation of the participants’ personal experiences, amicability, and if the participant perceived the CHW to behave or present him-or herself in a culturally appropriate manner (Table 2).

**Table 2:** Characterization of CHW relationship building

Alliance strategy	Evidenced participant quotation
Self-disclosure	“She even told me about her mother. As a friend, I have had much trust in her.” *Female, age 42, Spanish interview*
Offers validation	“Because I knew she was being honest and she was telling me the truth; she was giving me good advice” *Female, age 65, Spanish interview*
Perceived amicability	“Well, just like I was saying, she had an interest in us” *Female, age 71, Spanish interview*
	“The CHW would tell me, “Whenever you want, you are welcome in the group.”” *Female, age 31, Spanish interview*
Genuineness	“She was knowledgeable. She knew what she was talking about. She seemed interested, you know, to help me out. Not just one of those people who just come and give you the facts.” *Female, age 30, English interview*
Perceived desire to help	“She was helpful, direct, to the point, and friendly. It was her way of being and her attention to me.” *Female, age 45, Spanish interview*

Participants discussed perceptions of the CHWs’ professionalism, including how they demonstrated skills and appeared prepared to do their jobs by knowing the content well and by communicating messages about the content and its benefits. Participants also saw the CHWs’ professionalism as related to their efficiency and adaptability in carrying out their required role. Table 3 presents the results of a narrative analysis of respondents’ comments on CHWs’ professionalism.

**Table 3:** Elements of CHW professionalism

CHW characteristic	Evidenced participant quotation
Adaptability	“Yes. She had a very nice personality, she was very nice, very willing, because sometimes I couldn’t [meet] due to my children’s school and they would adapt themselves to my schedule.” *Female, age 35, Spanish interview*
Demonstrated skill	“Through their behavior and how they handled themselves, how they worked, the procedures they followed, I could see they were skilled.” *Male, age 48, Spanish interview*
Preparedness for presentation	“She is a very professional person, and she would motivate you to make changes of your eating habits, exercises. She came very prepared to motivate you to make changes in general, behavioral things, physically, foodwise, and she would give you a lot of advice.” *Female, age 33, Spanish interview*
Mirrors Messages from Primary Care Provider	“It was very similar to what the doctor had told me. They [CHW] would tell me very similar things in regards to portions. It was very important to eat fruits and vegetables, to do exercise. It wasn’t just the diet; that one had to do exercise, at least to walk or run.” *Female, age 35, Spanish interview*
Person-centered learning techniques	“Sometimes you don’t understand something and you ask her over and over and she has the patience to answer you.” *Female, age 41, Spanish interview*
Reduces technological divide	“She was certain about what she was talking about, because she was explaining everything on the computer or she would bring those pamphlets and tell us, “Look, follow this.” She’d explain really well, very very well.” *Female, age 54, Spanish interview*

Of the CHW attributes, adaptability was unique in that it involved the articulated steps that a CHW was willing to take in order to meet the unique needs of each participant. Professionalism was also tied into the CHW’s use of technology (laptop computers or tablets enabled with specific intervention content) in the participant’s home. The use of technology contributed to participant’s perception of the CHW’s knowledge of the content material and his/her expertise in navigating the technology necessary to transmit health improvement messages. Adding to this perceived professionalism was that bringing technology into the participant’s home was regarded as a sign of respect. Here, technology brokered professionalism and credibility with the participant, which seemingly facilitated the CHW intervention. This perception was furthered when the CHW reflected messages of the primary care provider and used person-centered learning techniques, which were respectful, kind and sensitive.

Once a relationship was fostered by rapport-building efforts and professionalism, there were often results that related to behaviors and life features beyond physical activity and healthful food choices. For example, some participants remarked that they shared what they learned from the CHW with their families and now felt accountable for modeling those healthy eating and physical activity behaviors. Others remarked on a sense of personal ownership over their mood and the steps necessary to become mentally healthy.

### Outcomes of the CHW-participant relationship

The participants of this sub-study were not selected only because they had achieved positive behavior changes. However, their comments indicated whether or not they were working to establish the recommended lifestyle changes. For some, changes in mood, healthy eating, or physical activity were facilitated by the CHW through various activities. Most salient was the use of focused and supportive messages that conveyed tangible examples of how to implement small changes. These strategies for change were accompanied by the perception that the CHW’s role was to bring new learning to the participant. Participants perceived the advice, guidance, and support they received as a form of counseling. Often, these messages were reinforced within culturally acceptable norms and suggestions to seek additional support from within a family or the community.

Beyond behavior change, there were other changes that were attributed to CHW influence. (Table 4).

**Table 4:** Outcomes related to positive CHW-Participant relationships

Outcome	Evidenced participant quotation
Lasting behavioral impact	“I stopped with it and after a while I went back to it. I did start exercising more. I did start trying to make healthier choices for my children…foodwise. As far as the food changes, yes. My kids, they know that every meal we have we have vegetables.” *Female, age 30, English interview*
	“They taught me how to eat, to measure the portions, the vitamins that each type of food has, what I shouldn’t eat.” *Female, age 57, Spanish interview*
	“Yes, and I started walking sometimes, right outside…I’d walk all the way to the post office and back and then right here outside sometimes at night. That’s what I liked the most. It was practical information.” *Female, age 54, Spanish interview*
	“Yes, she always reinforced the idea”...She always said, “Don’t stop walking.” Besides, they would invite me to attend the exercise program. I said, “I don’t have the courage to go alone,” and she said, “If you wish, I will go with you. Just let me know when and I will go with you.” *Female, age 57, Spanish interview*
Generational effect	“Well, in fact, since I, as well as my child, have diabetes, these talks helped us both. She showed me how to buy more nutritional items, for example, that were sugar free.” *Female, age 42, Spanish interview*
	“Sometimes it’s impossible to go to Zumba because it’s far away, so I put some music on and start dancing with my daughter.” *Female, age 31, Spanish interview*
Participant gratitude	“In fact, when I was undergoing psychological therapy and spiritual therapy, everything because I had had a very difficult moment. And, they helped me, with their talks, with their company, with the classes that they would give me…They came like a godsend at that moment.” *Female, age 35, Spanish interview*
	“In fact, my glucose level went down thanks to their recommendations about how to cook my food.” *Female, age 65, Spanish interview*
Institutional trust	“Very good, really good, that’s why when you said you come from there, I agreed immediately. They have my utmost respect, and whenever I see *Tu Salud Si Cuenta*, I’ve been around there by the Consulate, where they set their post, and as soon as I see it I go see. So, I’m happy and thrilled with all these years that they have been conducting research. I don’t usually answer the phone, but yesterday as I was about to hang up and you mentioned who you were representing, I changed my mind and said, “Yes, of course.”” *Female, age 54, Spanish interview*

A positive outcome was the generational effect of the participant’s sharing with others the information to change behavior. This was most easily observed with those who experienced a positive relationship with the CHW. They remarked on transmitting their learning to other members of their family, typically to their children. This was also accompanied by a sentiment of gratitude as well as evidence that they had begun to replicate the intervention or model it in their own lives. Often, a sense of institutional trust was developed with the sponsoring program or affiliated community partners. These perceptions were typically insulated by the idea that the CHW and the participant had a relationship with a degree of genuineness, so that overall the benefit of the CHW messages remained firm even after the intervention ended. Overall, those participants who felt the program was successful attributed that success to their relationship with the CHWs.

### Possible therapeutic effects derived within the context of the relationship

The therapeutic effects observed by those who experienced difficulty with aspects of mood were remarkable, with many experiencing improvements. The CHW not only assisted the participant with identifying and labeling mood states but also intervened to address the negative mood state. The manner in which CHWs offered assistance had parallels to popular therapeutic modalities (Table 5).

**Table 5:** Therapeutic effects

Therapeutic modality	Evidenced participant quotation
Cognitive behavioral therapy	“The point is to always be aware of what our body is feeling because sometimes we don’t notice and you are getting better.” *Female, age 35, Spanish interview*
Thought replacement	“She helped me to be able to identify and be able to reject those situations (participant speaking about when she felt lonely or isolated).” *Female, age 58, Spanish interview*
Acceptance and commitment therapy	“She helped me to accept what was happening because I was the only one who didn’t. But [the CHW relationship] helped me a lot.” (participant sharing about her partner’s extra marital affair) *Female, age 31, Spanish interview*
Biofeedback	“She told me that [depression] could be managed. We can involve our mind with other things, as with exercise, just getting outdoors, getting up, washing our face, to always think positively.” *Female, age 58, Spanish interview*
Behavioral activation	“You forget about everything and focus only on the exercise and [when] that helps you release the stress and gets you out of a bad environment. She always recommended that I go out.” *Female, age 31, Spanish interview*
Distraction techniques	“She recommended that I distract myself with the children. That I take them out to the park, that I not spend time thinking.” *Female, age 35, Spanish interview*
Harm reduction	“I was like, you know what? I’m going to stop drinking soda, and maybe I’ll drink apple juice, which I didn’t know also contains a lot of sugar and stuff like that.” *Male, age 43, English interview*
Psycho-education	“Sometimes a lot of people who are depressed have a tendency to gain weight or let themselves go and that always motivated us; exercising is good for many things, like overcoming a depression.” *Female, age 33, Spanish interview*
Positive self-talk	“I told her I felt very good because I followed all her indications and the diets, the size of the servings, and all that, right? And, I also put a great effort into it, and I saw everything was going well.” *Female, age 71, Spanish interview*
Constant positive regard	“Like when I was sad or something or she’d see me looking sad, she would always ask me if I was all right,” and she’d tell me, “don’t worry, you’ll get through it,” just motivating me, during my problems. *Female, age 39, Spanish interview*
Processing emotions	“Well, one can open up more to them and we feel more confident in being able to express…maybe even discuss those problems that are not even part of the program, but they would listen and help us, and it helped me a lot …and well, I would like to do it again.” *Female, age 58, Spanish interview*

These therapeutic effects included strategies that are rooted in aspects of thought replacement, distraction techniques, biofeedback, behavioral activation, and cognitive restructuring typical in acceptance and commitment therapy and cognitive behavioral therapy. It is important to note that these approaches originated from the CHW even though there was not specific counseling training, other than motivational interviewing provided. Overall, impact of the CHW’s work with participants was evident when they expressed confidence in the change process.

### Participants who did not experience change in depressive or anxious mood states

We did not expect that everyone would integrate or be receptive to the intervention. We saw 7 participants who experienced no change in either depressive or anxious mood states, as measured by psychometric scoring. The qualitative data for these individuals also signaled that they were less receptive to the CHW-delivered intervention and relationship. The evidence of how this is manifested in participant mood states is highlighted in Table 6.

**Table 6:** Degree of Depression or Anxiety Limiting Intervention Impact

Mood symptom	Evidenced participant quotation
Acculturative stress	*Interviewer:* “Did your CHW help you identify patterns in your behavior when you had a tendency to focus on negative thoughts, sadness or worry?” *Participant* “How can I explain, yes, worry, for me is that I’m in a country that’s not my own, I don’t speak English.” *Female, age 52, Spanish interview*
Participant limits intervention	“Yes, she insisted a lot, and I said I would start and because she kept coming I kept telling her I would start, and I never did. I didn’t follow through.” *Female, age 69, Spanish interview*
	“Actually, I haven’t told her, or she hasn’t asked about it.” *Female, age 29, Spanish interview*
Cognitive distortion	“Because when you are a person who is overweight you get embarrassed. I’m very shy and I get embarrassed. Because, sometimes people like doctors put you down, make you feel bad…I [would] rather do it on my own; that’s just the way I am.” *Female, age 30, Spanish interview*
Self- imposed isolation	*Interviewer:* “You feel overwhelmed sometimes with a lot of people?” *Participant:* “I do, I can’t stand it.” *Female, age 29, Spanish interview*
	*Interviewer:* “What do you think was preventing you?”
	*Participant:* “Probably myself.” *Female, age 30, Spanish interview*
Inappropriate guilt	“Today it feels like one of those days, depressing. When I’m having one of those days I say, ‘If I had done this,’ maybe this wouldn’t have happened.” *Female, age 31, Spanish interview*
Physical limitations	“I used to go to my yard and dig up the plants. I start to think about all that and say, ‘I can no longer do any of that,’ and I get sad.” *Female, age 61, Spanish interview*
Somatic complaints	“And it hurts. I can just be standing there, washing a plate, and my calves really hurt. I don’t know, it’s as if I had a bucket of cement in there and I can’t move it.” *Female, age 61, Spanish interview*
Unresolved grief and loss	“One reason is because my mom died at Christmas and the other…because my grandparents used to come for dinner for Thanksgiving. My mom died when she was 56 years old.” *Female, age 61, Spanish interview*

For some, negative mood states were related to influences that present cultural barriers to change, such as not speaking English. Others remarked on limiting the CHW relationship in some fashion, which reinforced their resistance to the intervention. Still others directly attributed their lack of behavior change to their level of malady. This was most clearly seen in their isolative behavior or negative self-talk. Lastly, some did remark that there were physical limitations that prevented the intervention from effecting change. These participants were discontented that the CHW relationship was unable to address their main concerns (e.g., not being listened to, being asked to do things that were embarrassing or uncomfortable). Consequently, those with consistently poor depression and anxiety scores tended to have negative or neutral reactions to the social/relationship nature of CHW visits.

### Participants with labile scores

Half of the participants (*n* = 11) had labile scoring patterns of depression and anxiety. That is, they experienced changes in their self-reported mood state throughout the course of the intervention. Of the labile scorers, 7 were identified with comorbid depression and anxiety, while the remaining 3 were labile in only depression, none in anxiety only, and 1 presented with mixed scoring for both anxiety and depression.

We analyzed labile scorers closer because they could offer insight into whether the CHW could improve mood states. Additionally, this group offered insight into the qualitative differences among those with improved mood. The following comparisons focused on the differences between monotonic increasing versus monotonic decreasing scorers.

### Comparison of labile scorers

All labile scorers (monotonic increasing or monotonic decreasing for measures of depression and anxiety) consistently commented that they had an amicable relationship with the CHW. Most often, CHWs were described as attentive, good listeners, supportive, personable, skilled and patient. This conveyed that participant’s perceptions of the CHW remained beneficial despite the outcomes of his/her mood states and self-reported behavioral outcomes. Regarding participants’ views of the intervention including the CHW relationship or their engagement or non-engagement in behavior change efforts (data not shown), we saw no qualitative differences in labile anxiety scorers, but there were differences among labile depression scorers related to their behavior change efforts (Table 7).

**Table 7:** Differences in labile depression scorers

Labile scoring group	Construct	Evidenced participant quotation
Monotonic increasing	CHW Feedback Mirrors Physician Messages	“These were changes that I was already carrying out upon recommendation of my doctor.” *Male, age 48. Spanish interview*
Monotonic increasing	CHW Feedback Aligned with Participant Effort	“I saw changes in my person. I began to feel better and that motivated me to continue doing [exercise and healthy eating]. I have been following this routine for 3 years and if 1 day I don’t do it, I feel like I am missing something.” *Male, age 64, English interview*
		“I followed her indications…and I also put great effort into it. I saw everything was going well. I mean, everything started to change because now I feel much lighter.” *Female, age 71, Spanish interview*
Monotonic increasing	CHW Support Tied To Improved Measureable Health Outcomes	“In fact my glucose level went down thanks to their recommendations about how to cook my food.” *Female, age 65, Spanish interview*
		“Motivation was the only thing that I needed, and I noticed some changes in that…I lost 15 pounds in 1 year.” *Male, age 55, Spanish interview*
Monotonic decreasing	Participant Reticence to Engage with CHW	“She would dedicate herself only to the topic…she wouldn’t talk about other things, she was only on the topic that she came to explain.” *Female, age 45, Spanish interview*
		*Interviewer:* “Were the changes you made as a result of the promotora?”
		*Participant*: “No, I was already making changes.” Female, age 39, Spanish interview
Monotonic decreasing	Comorbid Depression is Barrier to Relationship with CHW	“When I became unemployed, I got very depressed here in my house.” *Female, age 42, Spanish interview*
		“I try to get out of that [depressed mood]…but, it was awful.” *Female, age 45, Spanish interview*
		“When [CHWs] came by I was very depressed and so they told me, ‘You need to walk more, if you walk at least 15 min you will reject the anxiety a bit.’” *Female, age 41, Spanish interview*

In fact, while all participants were asked the same questions, the responses and constructs discussed by the 11 monotonic increasing and monotonic decreasing participants revealed different and distinct views. Those whose depression scores improved commented that the CHW mirrored health-related messages they had previously received from primary care providers. Also these participants more freely shared instances of positive behavior change as a result of the CHW, which also motivated persistence in the change process. Additionally, they commented that the CHW’s positive messages alone were not predictors of behavior change, that personal effort was also an integral component. This hinted at a greater awareness of the overall components of health for those in the monotonic increasing group. This may also signal a relationship between mood state and the capacity to be receptive to health messages, from the participant’s perspective.

The monotonic decreasing group acknowledged that the CHW was supportive of their change process but was not the source or the impetus for change. They indicated that their mood state (depression in particular) was overwhelming and suppressed engagement in the intervention. The participant’s awareness of comorbid depression appears to also play a role in the experience of no anxiety improvement.

It is important to note that all participants in the labile group had equal access to CHWs and their behavioral interventions, and characteristically, it was the monotonic increasing group that utilized, integrated and accepted the interventions in a way that allowed them to adopt improved health behaviors. Comparatively, this same group all commented on such measureable improvements in health outcomes as weight loss and improved blood glucose levels. Here, evidence of change behavior is linked to supportive CHW messages and their improvement in interventions outcomes and mood improvements. It appears that, overall, the monotonic decreasing group acknowledged comorbid depression but the monotonic increasing group did not although both groups’ levels of depression were verified by psychometric scoring. This may be partially attributed to the clinical impact of depression in that it often paralyzes individual action and prevents one from making behavior or cognition changes.

## Discussion

This study provides qualitative insight into participants’ perceptions of their interactions with CHWs who delivered a home visit–based intervention to understand the dynamics of the relationship and behavior change, particularly among participants who reported anxiety and depression measured by standard instruments. The study provides descriptive examples of rapport-building abilities used by CHWs to create close and beneficent relationships with participants, and our findings align with past research documenting beneficial CHW relationships [13, 17, 63, 64]. Also, this study presents the dynamics of these relationships by describing outcomes that include and surpass the targeted behaviors. Finally, the study explored therapeutic approaches that CHWs employed and the perceptions of participants who had anxiety and depression of the relationship and any benefits from the relationship.

The TSSC intervention delivered by CHWs was carefully designed to discuss physical activity and healthful food choices. However, within the intervention context, our study showed that not only did CHWs appear in many cases to influence change in lifestyle behaviors but they also influenced other positive changes, such as creating lifestyle changes across generations and creating institutional trust. Discussions between CHWs and participants revealed that change was influenced by the CHW who was professional and had strong rapport-building skills.

Prior to delivering the TSSC intervention, CHWs received motivational interviewing training to assist in lifestyle behavior change efforts but no other therapeutic approach training. Despite this, the CHWs influenced behavioral and mood changes and patterned their approaches after commonly used therapeutic techniques provided in a counseling psychotherapy training setting, and, as expected, many CHW messages were aligned with motivational interviewing. However, some approaches were more aligned with behavioral activation and cognitive behavioral techniques, that is, suggestions or activities that served to mitigate the negative mood state.

These findings highlight the specific potential to shift some counseling tasks to CHWs in terms of delivering behavioral health services, especially for the monotonic increasing group. Here, participants, with CHW support, demonstrated efficacy and agency to enact change behaviors. This is shown in their use of “change talk,” which is typically associated with motivational based psychotherapeutic behavioral interventions and behavior change. Even with this small sample size, replication and further examination of the task shifting concept may be appropriate, particularly when focused on the nuances of how co-occurring depression and anxiety are investigated in culturally specific community samples.

Our study expands the dearth of literature and bolsters the growing social and political support in the United States for the CHW serving as lay counselors and part of a healthcare team. Stacciraini’s [65] review examined research on the roles and outcomes associated with CHW-delivered interventions for mental health published in English, Spanish and Portuguese literature. The results revealed, particularly in the English literature, that at the time of the review a lack of rigorous study designs made conclusions about improved mental health outcomes unremarkable. Since then Tran et al. [66] reported findings from a pre-post test, pilot study where Latina community health workers intervened with Latinas expressing a history with mental health conditions and found improved depressive symptoms as post-test. Patel [67] has written extensively on the idea of task shifting in conjunction with meeting World Health Organization’s flagship program, the Mental Health Gap Program. His randomized controlled trial based in Brazil demonstrated that CHWs as compared to trained counselors were equally effective in improving depression scores of patients. Lawn et al. [68] found that lay support workers were an important part of an Australian care team and contributed to motivating complex patients to achieve behavioral change outcomes. Eli et al. [69] conducted a randomized trial with Latino patients with evaluated depression and either heart disease or diabetes. Usual clinical care was compared to lay promotoras providing psychoeducational sessions for 6 weeks followed by booster sessions. Results indicated that both intervention groups performed equally well across multiple measures including improvements in depression. Perhaps, as rigorous research of this kind continues it could contribute to alleviating the already-large deficit of mental health professionals and to building the capacity of an eager workforce to address community mental health service needs.

A strength of this study was selecting participants based on their anxiety and depression scores post-intervention. By doing this, we were able to characterize some ways that participants perceived the CHWs’ counseling strategies supported improved mood states, even with little specific training, no guidance from a counseling professional, and delivering an intervention focused on lifestyle changes, not necessarily mental health outcomes.

Additionally, the fact that we included participants who achieved positive behavior and mood change and those who did not allows greater insight into the roles of CHWs. The comments reflecting dissatisfaction with the CHW relationship and intervention provides important information to other researchers interested in similar questions. Further, this study qualitatively addressed the impact of the purposive sample stratification as a way to understanding the constructs that make up participant perceptions of CHW-assisted success in a behavioral intervention.

CHW-participant encounter time during the intervention was relatively short, perhaps similar to that of counseling sessions delivered in professional settings (once monthly for 6 months). Like the counseling setting, lack of resources limits the time intervention/counseling services can be provided, knowing that systemic and life circumstances may continue and may contribute to ongoing anxiety and depression. CHWs who delivered this intervention were trained to provide referrals to free or reduced-cost social services. These services, however, are limited for poorer communities, like the one from which our sample was drawn. Further, our participants largely did not have health insurance so they would, most likely, have unmet health and social service needs as would other members of their community.

A shortcoming of this study could be the limited demographic, social, and historical information collected. Therefore, we could not characterize influences such as country of birth, family size and support, or past trauma, all of which may have contributed to a richer understanding of the relationship between participants and CHWs. Our study did not assess to what extent the participant’s desire to please the interviewer may have influenced his/her positive description of the CHW interaction. Our results are not generalizable to all Latinos participating in lifestyle interventions, and further, our sample had few male participants. However, given the quality control measures we took with these data, we feel sure that some, though not all, bias was mitigated. Quality control measures included fidelity checks of the developed codes and themes as well as the overall construction of grounded theory. Verification checks were also done in data management and analysis to ensure integrity, especially as it related to participant’s quoted statements.

Future research may focus on whether knowing a participant’s scores on anxiety and depression instruments would influence CHW-participant relationships. In this study, the CHWs did not have that information, but had they known a participant’s anxiety and depression score, it may have influenced their approach, including continuing to support lifestyle changes despite the participant’s poor mood states or the CHW’s own frustration over lack of behavioral change. More research into the dynamics of the CHW-participant relationship and awareness of mood states is warranted, particularly because we found that labile mood states in this sub-study influenced some outcomes.

Further studies could also assess whether the role of CHW as lay counselor could be developed or amplified, when intentionally paired with physician or other healthcare professional messages. Likewise, the presence of comorbid depression appeared to play some role in the lack of improved anxiety. This could be due to several factors; however, participants were characteristically more open to sharing aspects of sadness and depression than of worry or anxiety. This could be cultural or it could be a lack of sensitivity and specificity in capturing these particular sub-populations’ perceptions of anxiety. Although we are not able to determine etiology, but because our purposive sample provided some insight, expressed comorbid depression and anxiety may warrant further study in this population.

## Conclusions

We undertook this study to characterize Latino participants’ perceptions of their interactions with CHWs who delivered a community intervention designed to understand the dynamics of that relationship and of behavior change. We provided insight into what facilitates a close relationship between a participant and a CHW and the behavioral change process. We saw that participants with consistently good anxiety and depression scores were open to developing relationships with CHWs who had strong rapport skills and professionalism and that the relationship supported behavior change. We also characterized how the CHW relationship may or may not influence participants who were experiencing anxiety and depression and how those persistent mood states manifested resistance to engaging in behavioral change interventions, especially as it related to participant learning.

While we saw that anxiety and depression had negative effects on the behavior change process, in this study most, but not all, participants who reported a negative mood state found that, to some extent, the CHW relationship beneficial in altering that state. Based on our qualitative evidence, therefore, we posit that task shifting should be tested further to determine its viability in providing counseling services to Latino populations in areas where mental health care services are limited or lacking.

## Appendix

**Table 8:** Semi structured interview guide questions

1. Please describe the relationship you had with your CHW.
2. Did your CHW help you make personal behavior changes, like beginning to exercise, portion control, or increasing the amount or fruits and vegetables you eat? If yes, how? If not, please comment?
3. Please describe the personal characteristics you found most and least helpful about your CHW.
4. Do you feel that your CHW had the skills to help you make personal behavior changes, like the ones listed above? If yes, why? If no, why not?
5. Did you trust in your CHW’s skills? If yes, please describe? If not, please comment?
6. Are the behavior changes you made as a result of the assistance of your CHW? If so, are these skills still in practice? How did you maintain these changes? If no, please comment.
7. Did your CHW help you identify sadness and worry in your life? Is so, how?
8. Did your CHW help you identify behaviors that made sadness and worry worse? If so, how?
9. Did you CHW help you gain skills to manage sadness or worry? If so, how?
10. Did your CHW help you identify patterns in your behavior where you tended to focus on negative thoughts, sadness or worry? If so, how?
11. Did you CHW help you identify where you tended to be more isolated or more social? If so, how?
12. Did your CHW help you in identifying where you were physically more active or inactive? If so, how?

## Abbreviations

BMI: Body mass index
CES-D: The Center for Epidemiological Studies Depression scale
CHW: Community health worker
IQR: Interquartile range
SAS: Zung Self-Rating Anxiety Scale
SD: Standard deviation
TSSC: *Tu Salud ¡Si Cuenta!*
